# Inflammatory Stress Response During Pregnancy as a Connecting Link in Intergenerational Risk Cascades

**DOI:** 10.1002/dev.70178

**Published:** 2026-06-30

**Authors:** Heidemarie K. Laurent, Sherryl H. Goodman, Karen D. Rudolph, Kento Suzuki, Penina Backer, Aastha Dhimal, Sarah Pikhit, Douglas A. Granger

**Affiliations:** ^1^ Department of Human Development and Family Studies Pennsylvania State University University Park Pennsylvania USA; ^2^ Department of Psychology Emory University Atlanta Georgia USA; ^3^ Department of Psychology University of Illinois Urbana‐Champaign Champaign Illinois USA; ^4^ Department of Biology Pennsylvania State University Harrisburg Pennsylvania USA; ^5^ Department of Biochemistry and Molecular Biology Pennsylvania State University University Park Pennsylvania USA; ^6^ Institute for Interdisciplinary Salivary Bioscience Research University of California Irvine Irvine California USA

**Keywords:** childhood maltreatment, obstetric outcomes, oral inflammation, pregnancy, stress response

## Abstract

Pregnancy represents a critical juncture for intergenerational transmission of stress‐related health vulnerabilities when child‐bearers’ history of maltreatment may precipitate stress responses that adversely impact maternal and fetal outcomes. We aimed to shed light on this path by testing hypotheses that (1) childhood maltreatment would predict oral inflammatory responses to acute stress in late pregnancy, and (2) these inflammatory responses would relate to subsequent obstetric complications. Hypotheses were tested in a community sample of women (*n* = 158) from a larger longitudinal study. Participants reported a history of maltreatment and completed the Trier Social Stress Test during the third trimester of pregnancy. Five saliva samples collected before and after the task were assayed for pro‐inflammatory cytokines (IL1β, IL6, TNFα) and C‐reactive protein (CRP) to index oral inflammation. At 3 months postnatal, women reported pregnancy and birth complications, and birth outcomes. Multilevel models of inflammatory response trajectories supported hypothesized paths: Greater childhood maltreatment—especially physical neglect and sexual abuse—predicted higher and/or extended inflammatory stress responses. In turn, higher inflammatory stress responses related to greater pregnancy and birth complications, and extended inflammatory responses related to a greater likelihood of preterm birth. Findings support oral inflammatory response to stress as a potential link to target in intergenerational health risk transmission.

## 1 Introduction

Effects of adversity are known to carry across generations, such that children born to parents who have themselves experienced high stress or adversity are more likely to develop health conditions that interfere with their own ability to cope with stress and care for themselves and others (e.g., Bowers and Yehuda 2016; Moog et al. 2021). The perinatal period represents a critical juncture in this chain of transmission when the fetus/infant is particularly susceptible to formative influences, and the accumulated stresses of the pregnant person may have the most pronounced effects on offspring. Toward explaining this phenomenon, researchers have proposed that prior (childhood) and/or concurrent (perinatal) stress disrupts the usual anti‐inflammatory protective environment during pregnancy, leading to poorer pregnancy and birth outcomes and ultimately the transmission of stress‐related health risks (e.g., Han et al. 2021). This conceptual model represents a specific application of the framework laid out by Tsuyuki et al. (2019)—based on toxic stress (Shonkoff et al. 2012) and social stress (Aneshensel 1992) theories—positing that early and ongoing stress experiences can perpetuate health problems across development by disrupting stress‐responsive physiological systems. However, knowledge regarding the details of this path in humans remains limited, restricting our understanding of how early life adversity shapes stress responsiveness during pregnancy, and how such response patterns may go on to impact the course of the pregnancy and childbirth. In this study, we aimed to shed light on this process by investigating key proposed links in this risk transmission chain: (1) associations between a history of adversity (childhood maltreatment) and pregnant women's inflammatory responses to acute psychosocial stress, measured in saliva, and (2) associations between their inflammatory stress response and quality of pregnancy and birth outcomes.

### 1.1 The Immune System Response to Stress and Variations in Pregnancy

As part of the human response to stress or challenge, activation of the sympathetic nervous system can set off a neurophysiological cascade resulting in central and peripheral production of pro‐inflammatory cytokines such as interleukin 1 beta (IL1β), interleukin 6 (IL6), and tumor necrosis factor alpha (TNFα), as well as downstream release of the acute‐phase protein C‐reactive protein (CRP). Together, these inflammatory mediators (IMs) are designed to protect the organism from pathogens, though excessive or prolonged inflammation has been implicated in a variety of health problems (Chavda et al. 2024). Meta‐analytic work has confirmed that acute psychological stressors increase levels of natural immune system indicators, including IL6, whereas prolonged or chronic stress results in diminished functionality of natural and specific immune system parameters (Segerstrom and Miller 2004). At the same time, significant heterogeneity in effect sizes points to individual differences requiring further investigation to understand health‐related risks across the life course.

A life phase known to profoundly alter immune system functioning is pregnancy. Over the course of a normal human pregnancy, shifts in maternal hormones typically orchestrate a progression from an initial pro‐inflammatory stage facilitating embryo implantation and placentation in the first trimester, followed by the downregulation of IMs and an anti‐inflammatory environment that supports healthy fetal development across the second and third trimesters and on‐time (as opposed to premature) birth, and finally to a pro‐inflammatory shift at the very end of the third trimester, triggering delivery (Mor et al. 2017; Robinson and Klein 2012). At the same time, individual differences in IM activity and stress responsiveness suggest that this protective shift is not universal—for example, it may be disrupted in women experiencing significant affective distress (Laurent et al. 2025)—and it is important to identify predisposing factors that maintain heightened inflammation across pregnancy and, in turn, precipitate risks to mother and fetus.

### 1.2 Effects of Early Adversity on Inflammation During Pregnancy

Available evidence for the effects of prior stress or adversity on inflammation during pregnancy is mixed. On the one hand, general stress based on racial status or acculturation has been associated with elevated levels of IL6 in pregnant women across their pregnancy (Scholaske et al. 2019) and in response to an acute psychosocial stressor (Christian et al. 2013). Another study documented higher levels of TNFα in pregnant women with a trauma history but no difference in IL6 levels (Blackmore et al. 2011). When early life adversity specifically has been examined, some researchers have detected effects of childhood maltreatment (physical and emotional abuse and emotional neglect, but not sexual abuse or physical neglect) on pregnant women's CRP levels, though they found no effects on IL6 or TNFα (Mitchell et al. 2018). Others have found no effect of early life stress on pregnant women's CRP but have found effects involving other markers of inflammation (i.e., pro‐inflammatory gene expression, IL6 levels; Leal et al. 2023; Walsh et al. 2016). Beyond differences in study sample characteristics and gestational timing, inconsistent or null results may have to do with relying on measures of inflammation outside of a stress context, as was true for the majority of studies cited here. If markers of earlier adversity are particularly apparent when confronting a stressor, it may be important to test childhood maltreatment effects in such a context.

To tap into acute stress response and recovery patterns, more easily collected and noninvasive salivary measures (as opposed to blood measures in the above studies) may be used. Although the degree to which salivary measures reflect systemic inflammation has been debated and may vary across IMs (Engeland et al. 2019; Slavish et al. 2015), there is good evidence that differences in oral inflammation relate in meaningful ways to psychological risk and well‐being, including among pregnant women (e.g., Laurent et al. 2025; Wong et al. 2023; Yui et al. 2022). To our knowledge, the only studies thus far addressing the effects of childhood adversity on oral inflammation have been conducted with nonpregnant samples and have similarly revealed mixed findings. Recent maltreatment was shown to predict elevated salivary CRP in young girls, but not boys (Entringer et al. 2020). In a general sample of adults, childhood maltreatment was not found to relate to individual salivary cytokines IL1β, IL6, or TNFα (Tollenaar et al. 2022), though another study in parents found that childhood adversity predicted higher levels of an inflammatory composite measure (Huffhines et al. 2021).

In the context of acute laboratory stress, there is a small body of emerging evidence supporting links between childhood adversity and inflammation. Preschool children's general stress was shown to predict higher levels of salivary IL1β (Tyrka et al. 2015), and childhood physical neglect was associated with adults’ IL1β gene expression and (blood) IL6 reactivity (Schreier et al. 2020). Thus, there are grounds for anticipating that childhood maltreatment could result in elevated inflammatory responses to later stressors, perhaps particularly for women and/or parents, though this has not yet been shown in pregnancy nor for all types of IMs. To determine if the first part of the proposed intergenerational risk chain is present, a comprehensive investigation of stress‐induced IMs in pregnant women is required.

### 1.3 Effects of Inflammation During Pregnancy on Obstetric Outcomes

The second part of the proposed chain would link elevated stress‐induced inflammation to adverse pregnancy and birth outcomes, and ultimately to offspring health risks; this has also not yet been demonstrated. There is robust evidence for a path from maternal systemic inflammation to processes that impair fetal development and precipitate complications like preeclampsia and preterm birth (e.g., Boyle et al. 2017; Habelrih et al. 2024; Harmon et al. 2016). None of this research directly addresses stress‐induced inflammatory processes, though heightened inflammation is thought to play a role in stress‐related disparities in pregnancy and birth outcomes (Giurgescu et al. 2016; Wadhwa et al. 2012). Studies of oral health problems in pregnancy show that oral inflammation—which, as noted above, can more easily be used to tap into stress responsivity—predicts some of these same obstetric complications. As discussed by Tsikouras et al. (2024), pregnant women with periodontal disease show a greater incidence of preeclampsia and preterm birth, with proposed mechanisms including a path from oral to systemic inflammation triggering harmful fetoplacental changes. In line with this explanation, a study of pregnant women with periapical dental infection documented effects on women's systemic inflammation, which in turn played a role in pregnancy length and infant size at birth (Harjunmaa et al. 2018). Together, this work suggests that further exploration of oral inflammation in relation to pregnancy and birth outcomes is merited. To identify and intervene on risky perinatal inflammation profiles early on, it is critical to determine whether stress‐induced salivary inflammatory responses are associated with complications of pregnancy and/or childbirth.

### 1.4 The Present Study

The present study was designed to gain an initial view of the proposed links between early adversity, stress‐related inflammation during pregnancy, and women's pregnancy/birth outcomes, with the ultimate goal of better understanding and intervening on intergenerational risk paths. To do so, we tested associations between self‐reported history of childhood maltreatment and salivary measures of inflammation (IL6, IL1β, TNFα, and CRP) in response to a psychosocial stressor in a community sample of women in their third trimester of pregnancy. We further tested associations between their (prenatal) salivary inflammatory responses and later (postnatal) reported obstetric complications. Based on the available evidence reviewed above and tenets of toxic stress theory (Shonkoff et al. 2012), we hypothesized that (1) history of maltreatment (abuse and/or neglect) would relate to higher and/or extended prenatal salivary inflammatory responses to stress and (2) higher and/or extended prenatal salivary inflammatory responses would be associated with greater complications of pregnancy and childbirth.

## 2 Method

### 2.1 Participants

Participants were drawn from a larger longitudinal study of mother–infant stress development. The sample was recruited from local health centers and community agencies serving pregnant people, as well as through social media advertising. To be eligible, participants had to be adult English speakers, expecting a singleton birth with no health complications that would preclude participating in stress tasks, and willing/able to complete a mix of remote and in‐person assessments until their child was 2 years old. The latter required living within an approximately 1‐h travel radius of the research lab at which the PI was located (central IL from T1 dates November 2017 to September 2020; central PA from T1 dates June 2022 to June 2024).

One hundred fifty‐eight participants completed the first assessment, ranging in age from 19 to 40 years (*M* = 30.98, *SD* = 4.92) and in gestational age from 19 to 40 weeks (*M* = 31.25, *SD* = 2.74). All participants self‐identified as women, and most (78%) were married. The majority identified as White European‐American (75.8%), followed by Black/African (10.8%), Latina (4.5%), Asian/Asian American (6.4%), and Multiracial (2.5%). Median reported income and education reflected middle‐upper socioeconomic status, though the observed range included those on the lower end (12% household income below poverty threshold, 17% had not completed college). One hundred twelve participants (71%) returned for the second assessment, at which obstetric information was collected. A comparison of those who continued in the study versus dropped out showed the former were older (*M* = 31.55 vs. 29.59 years, *t*[155] = 2.31, *p* = 0.022), later in their pregnancy at baseline (*M* gestational age = 31.58 vs. 30.44 weeks, *t*[156] = 2.43, *p* = 0.016), more educated (median = bachelor's degree vs. associate's degree, Mann–Whitney *U* = 3310.00, *p* = 0.002), and more likely to be married (84% vs. 65%, Wald *Z* = 2.57, *p* = 0.010). Continuers did not differ from dropouts on any other demographic variables, nor on childhood maltreatment variables. Tests for potential influence of these demographic variables revealed that while two of them—education and marital status—predicted women's oral inflammation levels, controlling for these factors did not meaningfully change the effects reported below. As described in the Analytic Strategy, the modeling approach used in this study allowed us to use the full sample who completed questionnaires at each time point to estimate associations with IM trajectories.

### 2.2 Procedures

The longitudinal study from which the present data are drawn consisted of five assessments with the pregnant mother and/or her infant: T1 was intended to occur during the third trimester of pregnancy, T2 at 3 months postnatal, T3 at 8 months postnatal, T4 at 15 months postnatal, and T5 at 24 months postnatal. Each assessment involved Qualtrics questionnaire completion, taking part in a psychosocial stress task and saliva sampling, and a clinical interview. All procedures were performed in compliance with ethical guidelines according to the Declaration of Helsinki and approved by the University of Illinois (protocol 18002 approved 8/12/2017) and/or Pennsylvania State University (protocol 19133 approved 2/8/2022) Institutional Review Boards. Participants gave written informed consent prior to taking part in the study procedures.

Participating women were scheduled for their first session either in the laboratory or remotely via Zoom as necessitated by COVID‐19 pandemic restrictions, and by participant preference following the lifting of such restrictions. All sessions were scheduled in the afternoon to reduce variability related to biomarker diurnal rhythms. Following written informed consent, participants completed pretask questionnaires covering information relevant to salivary stress measures; if the participant was sick with a fever, they were rescheduled, and other information relevant to oral and general health was considered in covariate testing as described below.

Following an initial saliva sample, participants were given instructions for the stress task, the Trier Social Stress Test (TSST; Kirschbaum et al. 1993; see Gunnar et al. [2021] for validation of an online version of the task). Following a 5‐min speech preparation period, the participant engaged in 5 min of public speaking and 5 min of mental arithmetic in front of a “judge” (trained research confederate). Immediately after the task, the second saliva sample was collected, after which the participant continued completing study questionnaires and/or the clinical interview (dependent on whether the latter could be scheduled during the session). The third saliva sample was collected 25 min after the start of the TSST, and the fourth and fifth samples were collected at 20‐min increments following the preceding sample. For each sample, the participant was asked to passively drool into a collection tube for 2 min (timed by the researcher). All samples were placed in a laboratory or home freezer before being transferred to a −60°C freezer for storage until they could be shipped on dry ice for assay at the Institute for Interdisciplinary Salivary Bioscience or Salimetrics Laboratory. These procedures are in keeping with current best practice recommendations for salivary IM measurement (see Riis et al. 2021) while allowing for home sample collection to accommodate remote sessions.

### 2.3 Measures

#### 2.3.1 Childhood Trauma Questionnaire—Short Form (CTQ‐SF; Bernstein and Fink 1998)

Maltreatment history was assessed at T1 with a widely used self‐assessment measure composed of five 5‐item subscales tapping Emotional and Physical Neglect and Emotional, Physical, and Sexual Abuse. Participants rated 25 items to indicate how often various experiences occurred during their childhood from 1 = *Never* to 5 = *Very Often*. Mean scores were computed for each subscale, and a summed score across all scales represented total maltreatment. Example items include “I knew there was someone to take care of me and protect me” (Physical Neglect, reverse coded) and “Someone molested me” (Sexual Abuse). In the current sample, subscales showed adequate internal consistency similar to metrics from validation studies with this measure (Cronbach's alpha = 0.91 for Emotional Neglect, 0.71 for Physical Neglect, 0.82 for Physical Abuse, 0.95 for Sexual Abuse, 0.88 for Emotional Abuse). A meaningful proportion of the sample scored in the elevated (moderate‐severe maltreatment) range according to the CTQ manual for each subscale: 12% for Emotional Neglect, 13% for Physical Neglect, 10% for Physical Abuse, 10% for Sexual Abuse, and 21% for Emotional Abuse.

#### 2.3.2 Obstetric Complications

As part of their first postpartum (T2) assessment, mothers were asked to report on their pregnancy and childbirth experiences. *Total pregnancy complications* was a summed score of mother‐endorsed gestational diabetes, high blood pressure, anemia, injury, infection, anxiety, depression, vaginal bleeding, severe nausea, cerclage, placenta previa or abruptio placentae, preterm labor, premature rupture of membranes, and steroid treatment. *Prematurity* was a binary variable based on birth at <37 weeks of gestation. *Birth weight* was a continuous variable representing infant pounds and ounces at birth. *Vaginal birth* was a binary variable based on reporting vaginal (as opposed to elective or emergency Caesarean) birth method. *Total birth complications* was a summed score of mother‐endorsed infant features, including blue at birth, slow heartbeat, not breathing, convulsions, needed oxygen, blood transfusion, an incubator, hypoglycemia, and a NICU stay. Whereas higher pregnancy and birth complications and prematurity variables indicated greater risk, higher birth weight and vaginal birth variables indicated lower risk outcomes. Similar obstetric risk composite scores have shown utility in predicting maternal/fetal morbidity (e.g., Chaalan et al. 2024; Easter et al. 2019).

#### 2.3.3 Salivary IMs

All samples were assayed in duplicate for IL1β, IL6, TNFα, and CRP, with averages of duplicate results used in analyses (see Riis et al. 2021). On the day of assay, samples were allowed to thaw to room temperature and centrifuged at 3500 rpm for 15 min to pellet remaining mucins. The Human CRP (Vascular Injury Panel 2) V‐Plex Meso Scale Discovery (MSD) multi‐spot Assay (Ref# K0080900) measured salivary CRP, with samples diluted fivefold (MSD Assay Diluent) prior to the assay. The Human ProInflammatory V‐Plex Meso Scale Discovery (MSD) multi‐spot Assay (Ref# K008074) measured cytokines, with samples diluted twofold (MSD Assay Diluent) prior to the assay. Salivary analyte concentrations (pg/mL) were determined with MSD Discovery Workbench Software (v. 4.0) using curve fit models (4‐PL with weighting function option of 1/*y* ^2^). MSD determines the detection limit for each analyte by interpolating lowest value on the standard curve plus 2.5 SDs (Avg Concentration_blank_ + 2.5 SD_blank_) over several kit lots. Intra‐ and interassay coefficients of variation were, on average, <10% and 15%, respectively (see also Martinez et al. 2018).

Per testing center recommendations, all biomarkers were screened for assay values outside of detection limits, and low values were substituted with half the lower limit and high values with the upper limit. In total, this impacted a relatively small proportion of sample values (0.26%–6.58% across biomarkers). Given the positive skew of biomarker distributions, all scores were natural log‐transformed for further analysis.

#### 2.3.4 Control Variables

Various factors potentially influencing the measurement of salivary biomarkers were assessed for influence. These included participant‐reported recent food/drink; tooth brushing, cavities, dental work, and oral injuries; medications and other substance use; sleep/wake times; and exercise. They also covered oral health habits (i.e., frequency of dental appointments and tooth brushing) and body mass index pre‐ and post‐pregnancy. Initial model testing to identify relevant controls suggested that participants’ current use of certain medications—antihistamines, thyroid medication, and antibiotics—was associated with lower levels of multiple IMs, and several other variables (BMI, recent oral injury or dental work, nicotine use within the past 24 h) showed associations with a single IM. Given the questionable value of including covariates that only applied to a small number (six or fewer) of participants, all models were tested both with and without control variables to determine whether their inclusion changed findings. No meaningful changes were detected—that is, same pattern of significant effects, <15% change in coefficient—for all primary model effects; therefore, the simpler models without covariates are reported below.

### 2.4 Analytic Strategy

Multilevel growth curve modeling using the HLM 7.0 program was selected to test hypotheses regarding individual differences associated with salivary IM response trajectories. This approach separates variability into within‐person (Level 1) and between‐person (Level 2) units to yield accurate standard errors for testing regression coefficients given the dependent data structure. Here, we modeled each mother's salivary IM values over time—both individual IMs and an IM composite averaging across standardized IM scores—at Level 1, then added individual difference variables at Level 2 to predict differences in IM trajectories. Because HLM uses full‐information maximum likelihood estimation to arrive at model parameters in the presence of missing data at Level 1, all participants who completed T1 (*n* = 158) were included in tests of CTQ‐inflammation effects, and all participants who completed T2 (*n* = 112) were included in tests of obstetric complication‐inflammation effects.

As described in Laurent et al. (2025), we had previously established that IM trajectories could be modeled as a quadratic function with an intercept centered on the third saliva sample (representing IM level following the stressor), linear slope (representing rate of IM reactivity or recovery following the stressor), and quadratic slope (representing overall steepness of the response trajectory across the session), all of which varied significantly across participants.

To test our primary research questions, CTQ childhood maltreatment (Question 1) or obstetric complication (Question 2) scores were entered as Level 2 predictors of mothers’ IM trajectory terms. A positive association between each of these risk characteristics and IM intercepts and/or linear slopes would support hypothesized effects. We note that for testing the second research question, this meant using measures collected later in time (i.e., T2 obstetric outcomes) to predict previously observed (T1) IM responses; this was done to leverage the advantages of multilevel modeling for testing associations between nested data structures of interest and individual difference characteristics. We confirmed that the pattern of effects reported using these models below was similar to that arrived at through simple linear regression tests, with participant‐specific HLM trajectory parameter estimates predicting obstetric outcomes.

## 3 Results

Descriptive information for study variables is shown in Table 1. Bivariate correlations were tested to provide information about associations among mother‐reported risk variables. These confirmed that different forms of maltreatment were related to one another (*r*s = 0.19–74, *M* = 0.47, *p* < 0.001–0.016 across CTQ subscales). Pregnancy complications were associated with maltreatment history (*r* = 0.40, *p* < 0.001 with total CTQ), though birth complications and infant birth weight were not (*r* = 0.09, *p* = 0.36 and *r* = −0.06, *p* = 0.53, respectively). Among obstetric outcomes, pregnancy complications related to birth complications (*r* = 0.33, *p* < 0.001) but not to birth weight (*r* = −0.16, *p* = 0.083), and birth complications related to lower birth weight (*r* = −0.19, *p* = 0.045).

**TABLE 1:** Mother‐reported risk variable descriptives.

Variable	Range	*M*, *SD* or %
CTQ		
Emotional Neglect	1–4.6	1.87, 0.88
Physical Neglect	1–3.6	1.35, 0.53
Physical Abuse	1–4.4	1.29, 0.55
Sexual Abuse	1–4.8	1.17, 0.57
Emotional Abuse	1–4.6	1.85, 0.95
Pregnancy complications	0–8	1.08, 1.38
Gestational diabetes		16%
High blood pressure/preeclampsia		10.6%
Anemia		10.6%
Serious injury		0.9%
Infectious disease		1.8%
Anxiety		24.8%
Depression		15.9%
Vaginal bleeding		6.2%
Severe nausea/vomiting		8.8%
Cerclage		0%
Placental problems		3.5%
Preterm labor		5.3%
Premature rupture of membranes		0.9%
Steroid medication		1.8%
Birth complications	0–5	0.47, 0.93
Baby blue at birth		4.4%
Slow heartbeat		3.5%
Not breathing		4.4%
Convulsions		0%
Needed oxygen		8.0%
Blood transfusion		0%
Needed incubator		6.2%
Hypoglycemia		9.7%
NICU stay		10.6%
Birth weight (lbs.)	4.69–9.81	7.44, 0.93
Vaginal birth		72%
Premature birth		4%

*Note:* CTQ subscale scores represent mean ratings.

**TABLE 2:** Multilevel model results—Association of childhood maltreatment with inflammatory mediator response trajectories.

	Predictor variable					
Outcome	Total maltreatment Coeff, SE	Physical neglect Coeff, SE	Emotional neglect Coeff, SE	Physical abuse Coeff, SE	Sexual abuse Coeff, SE	Emotional abuse Coeff, SE
IM composite						
Intercept	**0.16, 0.074**	*0.13, 0.069*			**0.17, 0.068**	
Linear slope	0.013, 0.013	**0.026, 0.012**			−0.010, 0.013	
Quadratic slope	−0.0041, 0.0072	−0.0040, 0.0074			0.00077, 0.0067	
IL6						
Intercept	**0.17, 0.074**	**0.15, 0.069**	**0.18, 0.079**			
Linear slope	0.012, 0.010	**0.020, 0.0096**	0.0013, 0.011			
Quadratic slope	−0.0014, 0.0061	−0.0018, 0.0065	0.00078, 0.0065			
IL1β						
Intercept					**0.16, 0.073**	
Linear slope					−0.015, 0.013	
Quadratic slope					0.0039, 0.0050	
TNFα						
Intercept				**0.15, 0.065**		
Linear slope				−0.0032, 0.012		
Quadratic slope				−0.0048, 0.0066		
CRP						
Intercept	**0.19, 0.068**	**0.14, 0.067**			**0.16, 0.059**	**0.17, 0.069**
Linear slope	0.014, 0.012	**0.024, 0.010**			0.0026, 0.011	0.017, 0.012
Quadratic slope	−0.0082, 0.0063	−0.0036, 0.0063			−0.00089, 0.0049	**−0.014, 0.0062**

*Note:* Results from models with one or more significant effects are shown; all coefficients are standardized values; significant effects (*p* < 0.05) are highlighted in bold; marginal effects (*p* < 0.10) are italicized.

**TABLE 3:** Multilevel model results—Association of obstetric complications with inflammatory mediator response trajectories.

	Predictor variable			
Outcome	Pregnancy complications Coeff, SE	Birth complications Coeff, SE	Vaginal birth Coeff, SE	Premature Coeff, SE
IM composite				
Intercept	**0.22, 0.066**			−0.37, 0.59
Linear slope	0.011, 0.017			**0.14, 0**.039
Quadratic slope	−0.0050, 0.0084			**−0.076, 0.020**
IL6				
Intercept	**0.16, 0.075**		−0.35, 0.23	0.012, 0.46
Linear slope	0.0067, 0.014		**−0.057, 0.028**	*0.12, 0.062*
Quadratic slope	−0.0091, 0.0072		−0.000023, 0.018	**−0.030, 0.015**
IL1β				
Intercept	**0.24, 0.076**	**0.18, 0.079**		−0.41, 0.56
Linear slope	0.010, 0.016	0.017, 0.017		**0.18, 0.042**
Quadratic slope	0.00042, 0.0072	**−0.019, 0.0057**		*−0.048, 0.028*
TNFα				
Intercept	**0.16, 0.071**			−0.31, 0.31
Linear slope	0.0074, 0.018			*0.10, 0.052*
Quadratic slope	−0.0020, 0.010			**−0.12, 0.049**
CRP				
Intercept			**−0.54, 0.20**	
Linear slope			−0.0085, 0.031	
Quadratic slope			0.0053, 0.018	

*Note:* Results from models with one or more significant effects are shown; all coefficients are standardized values; significant effects (*p* < 0.05) are highlighted in bold; marginal effects (*p* < 0.10) are italicized.

For primary model testing, quadratic HLM models were fit to the IM composite outcome, as well as to individual IMs, and mother‐specific predictors were added in separate models to explain between‐participant variability in intercept, linear, and quadratic slope terms.

### 3.1 Step 1: History of Childhood Maltreatment Related to Inflammatory Responses (Table 2; Figure 1)

To address our first research question, CTQ scores were tested as Level 2 predictors of mothers’ IM trajectories. The total maltreatment score significantly predicted higher IM composite intercepts; when individual IMs were examined, this association was significant for IL6 and CRP. Testing each form of maltreatment separately showed that greater physical neglect predicted more positive IM composite intercepts (marginal effect) and linear slopes (significant effect); both of these effects proved significant for IL6 and CRP measures individually. Greater sexual abuse also predicted higher IM composite intercepts, an effect that held for IL1β and CRP measures. More limited sets of associations in the same direction were found for greater emotional neglect (predicting higher IL6 intercepts), physical abuse (predicting higher TNFα intercepts), and emotional abuse (predicting higher CRP intercepts). Together, these results pointed to higher and/or more extended inflammatory responses during the stress task associated with greater childhood maltreatment.

**FIGURE 1:**
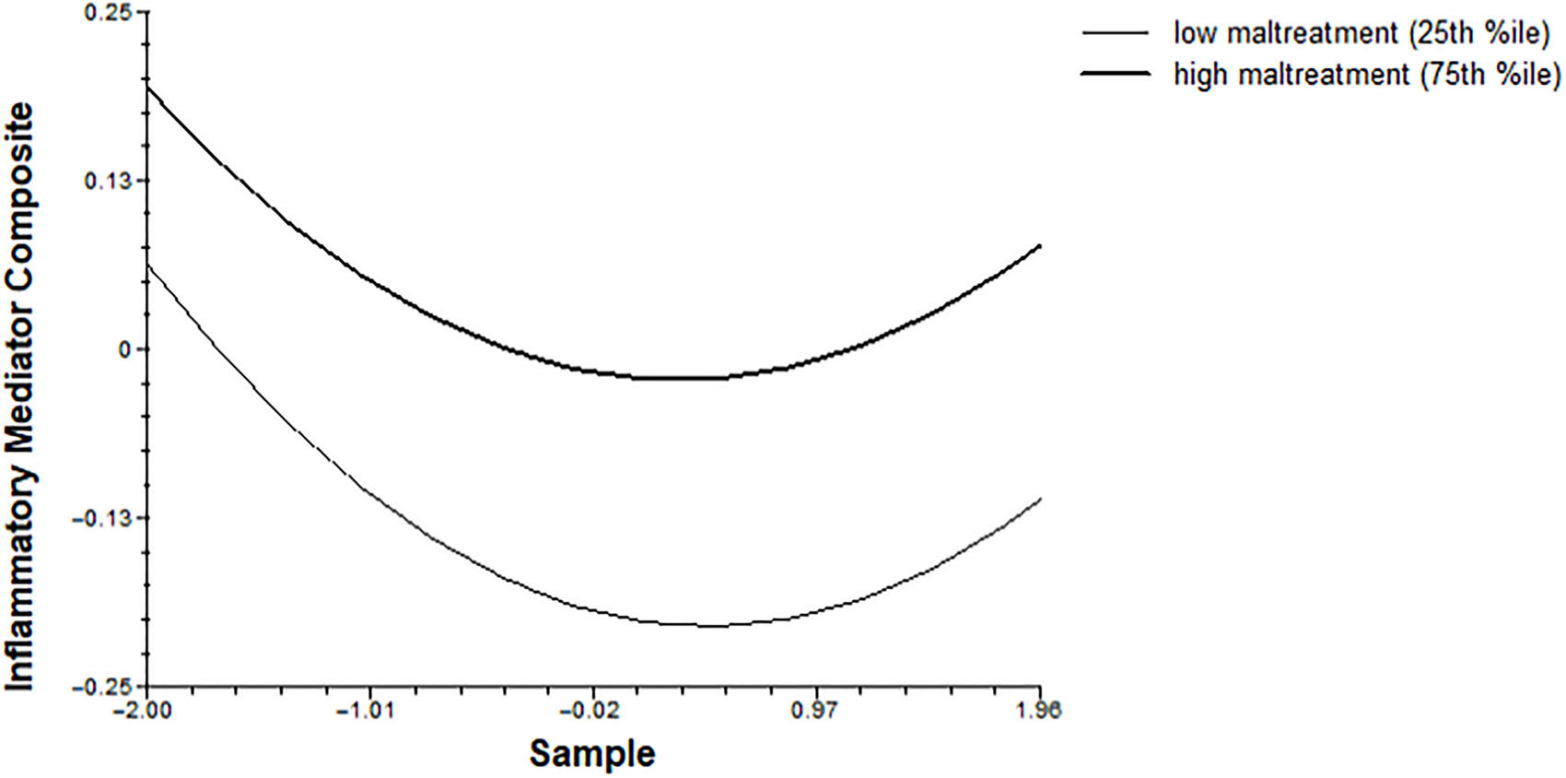
Total childhood maltreatment related to inflammatory mediator composite stress response trajectories.

### 3.2 Step 2: Inflammatory Responses Related to Obstetric Complications (Table 3; Figure 2)

To address our second research question, obstetric complication variables were tested as Level 2 predictors of IM trajectories. Total pregnancy complications showed a significant positive association with IM composite intercepts, an association that held for IL1β, IL6, and TNFα measures individually. Premature birth was associated with more positive IM composite linear slopes and more negative quadratic slopes; each of these effects was significant or marginally significant for IL1β, IL6, and TNFα measures. Higher total birth complications were associated with higher IL1β intercepts and more negative quadratic slopes. Finally, having a vaginal birth was associated with lower CRP intercepts and more negative IL6 linear slopes. Overall, these results converged to show that higher and/or more extended inflammatory stress responses during pregnancy were associated with greater obstetric complications across pregnancy and childbirth.

**FIGURE 2:**
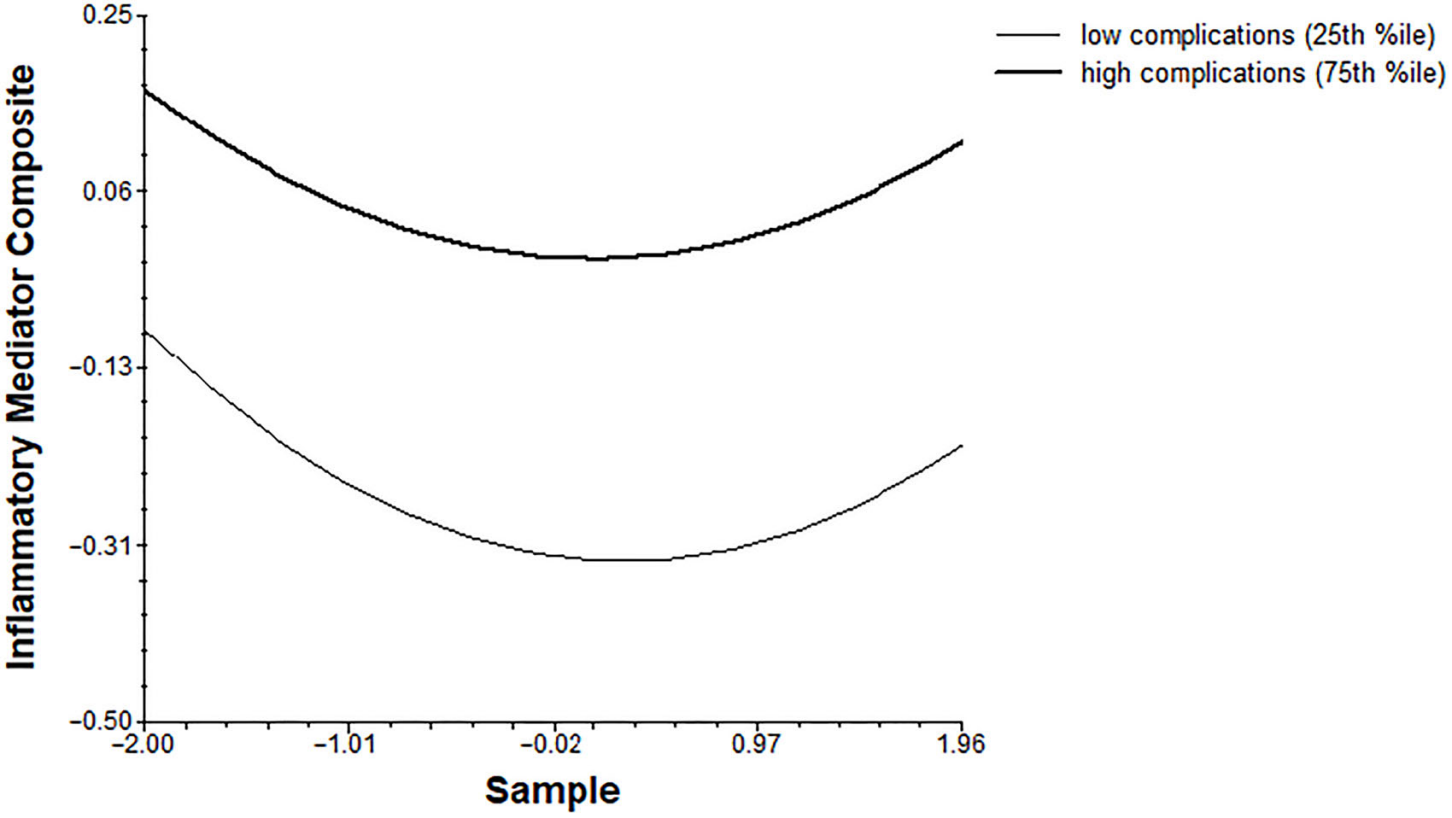
Total pregnancy complications related to inflammatory mediator composite stress response trajectories.

## 4 Discussion

In this study, we sought to determine whether early adversity predicted differences in women's salivary inflammatory response to stress during pregnancy, and whether such inflammatory stress profiles in turn were associated with obstetric complications that could impact their offspring's well‐being. Overall, we found support for hypothesized links between early adversity in the form of childhood maltreatment, inflammatory hyperresponse to stress during late pregnancy, and greater pregnancy/birth complications. Although preliminary, these findings support a prenatal inflammatory nexus in intergenerational risk transmission paths that could provide a target for family health prevention/intervention efforts.

In our community sample of pregnant women, a history of childhood maltreatment related to higher and/or increasing inflammatory response to acute stress, providing support for the first link in the proposed risk path. Although each form of maltreatment bore relation to some aspect of inflammatory hyperresponse, physical neglect and sexual abuse showed particularly widespread effects. The former predicted a higher, extended response trajectory for the inflammatory composite, as well as IL6 and CRP individually, and the latter predicted a higher response trajectory for the inflammatory composite, and IL1β and CRP individually. These findings echo previous research documenting higher IL6 and/or CRP (in blood or saliva) in individuals who had experienced some form of adversity (Christian et al. 2013), and maltreatment more specifically (Entringer et al. 2020; Schreier et al. 2020; Walsh et al. 2016). Together with these studies, the present results suggest childhood maltreatment of varying forms can result in both short‐ and long‐term increases in inflammation, at rest and in response to stress. These findings further echo and amplify a social stress theory account of lifelong effects of violence against women (i.e., through childhood abuse and/or later intimate partner violence) on health and disease via stress–immune dysregulation (Aneshensel 1992; Tsuyuki et al. 2019). Although a focus on specific type/s of maltreatment is likely premature given the mixed state of the literature, it is noteworthy that physical neglect emerged as a predictor of IL6 stress reactivity in both this study and a previous study of nonpregnant adults using blood measures (Schreier et al. 2020); such findings underline the importance of attending not only to experiences of threat or abuse but also to deprivation or neglect to identify and intervene in the development of a stress‐hyperresponsive inflammatory system.

Knowing that the consequences of elevated inflammation may be particularly far‐reaching during pregnancy, it is further critical to determine whether stress‐responsive inflammation relates to obstetric problems. The Walsh et al. (2016) study hinted at such an effect by showing an association between high IL6 in pregnancy and shorter gestational length, but this was only true under certain conditions (i.e., in cases without a history of severe abuse), and not in relation to stress, specifically. Addressing this second link in the proposed risk path, we demonstrated that differences in inflammatory stress response levels and/or dynamics related to a variety of obstetric outcomes. Specifically, women with higher levels of any of the inflammatory cytokines (IL6, IL1β, and TNFα) during the prenatal stressor reported a greater number of total pregnancy complications, and those with increasing inflammatory response dynamics (especially IL6 and IL1β) reported poorer birth outcomes, including a higher incidence of prematurity. This finding fits with and builds on evidence from periodontal disease research suggesting that oral inflammation—here, in response to stress specifically—heralds disruption in the normative progression of a healthy pregnancy, perhaps via systemic inflammation inducing premature birth (Tsikouras et al. 2024). The fact that medical complications of preterm birth have recently been shown to extend to a range of adult (age 35) mental and physical health problems underlines the importance of identifying and targeting factors that precipitate this outcome (see D'Agata et al. 2025).

The one hypothesized outcome for which we failed to find any significant effects was birth weight; this could be due to inaccuracy in retrospective reporting of this specific number and/or the limited proportion of high‐risk pregnancies dictated by our sample screening process upon enrollment. Future prospective research with a greater range of obstetric risk will be needed to fully test such effects. For now, our preliminary results highlight increased inflammation as a factor that may help to explain known stress‐related disparities in pregnancy and birth outcomes, in that women who are exposed to more stress and/or more reactive to such stress may experience a breakdown of the usual protective anti‐inflammatory environment, putting them and their fetus at risk for further health difficulties.

Of the different IMs examined, IL6 emerged as a prominent factor in links with both prior adversity and obstetric complications. As reviewed in Jones et al. (2018), the pleiotropic nature of IL6 allows it to not only play a key role in innate and adaptive immunity but also exert hormone‐like effects on homeostatic regulation of metabolic and neuroendocrine systems that, in turn, impact diverse health outcomes. Human and rodent models have implicated prolonged or excessive IL6 activity in adverse pregnancy and birth outcomes (i.e., triggering preterm birth, interfering with fetal brain development; Chang et al. 2023; Rudolph et al. 2018), and some researchers have suggested IL6 antagonists may be useful as a therapeutic measure to prevent such outcomes in high‐risk pregnancies (Côté et al. 2025). Our findings add context to understanding who may be most prone to IL6 hyperactivity during pregnancy—that is, women with a history of maltreatment who continue to experience high levels of psychosocial stress—and could be used to aid in screening efforts. For example, women who present for antenatal care with a history of maltreatment could have their IL6 levels monitored across pregnancy, with pharmacological or behavioral therapeutics offered to help lower as necessary.

Situating these findings more broadly within basic and applied research on inflammatory stress and intergenerational risk, our results provide evidence for main effects of parental risk factors that may be compounded by or transmitted through additional risks; next steps in this line of research should probe key moderators and mediators that can inform perinatal prevention/intervention strategies. For example, Huffhines et al. (2021) showed that the combination of childhood and later adversity predicted higher inflammation in mothers, as well as in their children, suggesting that the accumulation of adverse experiences over time should be assessed and flagged as women enter pregnancy to protect both themselves and their offspring. Mental health difficulties also may potentiate maltreatment effects—consistent with Walsh et al.’s (2016) finding of higher inflammation in pregnant women with both an abuse history and current depression—or serve as a mediator linking adverse experiences to inflammation, as implied by previously detected associations between affective distress and inflammatory stress hyperactivity in this sample (Laurent et al. 2025). Indeed, we found evidence of a reduction in the unique effects of childhood maltreatment on IMs in post hoc tests controlling for concurrent affective symptoms during pregnancy and, to a lesser extent, for a lifetime history of affective disorder diagnoses, suggesting that early maltreatment may give rise to inflammation in part through ongoing vulnerability to stress and distress across development. Of relevance, behavioral interventions developed to mitigate stress and distress, such as mindfulness training, have been shown to reduce salivary and systemic inflammation (e.g., Lengacher et al. 2018; Moreno 2024; Sanada et al. 2020). Better knowledge of the boundary conditions for when and how early adverse experiences precipitate high inflammatory stress would help to fine‐tune recommendations for who should receive such training during the perinatal period and—through randomized controlled trials—assess in a more controlled way what is required to interrupt risk transmission paths.

Going forward, the limitations of the present study should be used to guide research building on these findings. First, our sample was restricted in size and range of risk severity, limiting statistical power to detect effects. Our observation that participants who dropped out before the postnatal assessment were those most likely to show elevated inflammation—that is, based on the association between lower education and unmarried status and both dropout and salivary IM levels—underscores this limitation. To better maintain a diverse sample in such research, investment in a consistent and actively engaged point of contact for participants across the longitudinal study period is needed. For example, embedding a research coordinator within an established social services or healthcare agency with which women are continuously connected could help foster relationships that last throughout the ups and downs of pregnancy and new parenthood. The fact that significant effects we did find were small in size is consistent with adaptive features of the immune system (i.e., built‐in reserve capacity and redundant subsystems that can compensate for one another) and underscores the need for larger and more diverse samples to provide a clearer picture of associations involving more complex moderated or mediated paths.

Second, we only considered certain forms of prior adversity (childhood maltreatment), current stress responding (to the TSST), and obstetric outcomes (retrospective mother‐reported pregnancy and birth complications, prematurity, birth weight). These provided preliminary evidence of hypothesized links in larger risk paths but were limited in scope; future prospective studies should probe differential effects based on type/s and timing of adverse experiences, inflammatory responses (ideally in blood as well as saliva) to different types of stressors across pregnancy, and objective measures of a range of obstetric outcomes based on medical records. As noted previously, the degree to which salivary measures of inflammation reflect systemic inflammatory processes remains under debate, though the confluence of current findings with previous research using blood measures and significant effects involving salivary CRP (which has been more strongly associated with blood levels) bolster confidence in the relevance of our findings to the broader inflammatory stress literature.

Finally, although the present findings are consistent with a larger path model in which stress‐induced inflammation explains associations between childhood maltreatment and perinatal health complications, we were not powered—particularly given the exclusion criteria dictating a lower‐risk sample—to fully test such mediated paths. Post hoc probes of indirect effects via the Sobel test revealed marginally significant paths from childhood maltreatment to pregnancy complications via higher IM intercepts, laying a preliminary foundation for testing in larger samples representing a greater proportion of high‐risk pregnancies. Previous research further points to possible mechanistic paths underlying these associations and implications for infant development that we were unable to directly test here. Research designs that can tap these features through longitudinal investigation in both humans and nonhuman animals will be required to advance knowledge in these areas.

Limitations notwithstanding, the present study offers notable strengths as the first to our knowledge to (1) examine stress‐related inflammatory responses during pregnancy in relation to both upstream risks and downstream outcomes proposed by intergenerational risk transmission models and (2) include a comprehensive assessment of an array of IMs and modeling of not just levels but also dynamics of inflammatory stress response. With this work, we provide initial confirmatory evidence of an inflammatory stress link in the chain of stress risk transmission that could help guide efforts to interrupt such cascades.

## Funding

This research was funded by the National Institute of Child Health and Human Development (Grant No. R01HD093627).

## Ethics Statement

All procedures were performed in compliance with ethical guidelines according to the Declaration of Helsinki and approved by the University of Illinois (protocol 18002 approved 8/12/2017) and/or Pennsylvania State University (protocol 19133 approved 2/8/2022) Institutional Review Boards. Participants gave written informed consent prior to taking part in the study procedures.

## Conflicts of Interest

The authors declare no conflicts of interest.

## Data Availability

Deidentified data are available from the first author upon reasonable request.

## References

[dev70178-bib-0001] Aneshensel, C. S. 1992. “Social Stress: Theory and Research.” Annual Review of Sociology 18: 15–38. 10.1146/annurev.so.18.080192.000311.

[dev70178-bib-0002] Bernstein, D. P. , and L. Fink . 1998. Childhood Trauma Questionnaire: A Retrospective Self‐Report Manual. The Psychological Corporation.

[dev70178-bib-0003] Blackmore, E. R. , J. A. Moynihan , D. R. Rubinow , E. K. Pressman , M. Gilchrist , and T. G. O'Connor . 2011. “Psychiatric Symptoms and Proinflammatory Cytokines in Pregnancy.” Psychosomatic Medicine 73: 656–663. 10.1097/PSY.Ob013e31822fc277. 21949424 PMC3188677

[dev70178-bib-0004] Bowers, M. E. , and R. Yehuda . 2016. “Intergenerational Transmission of Stress in Humans.” Neuropsychopharmacology 41: 232–244. 10.1038/npp.2015.247. 26279078 PMC4677138

[dev70178-bib-0005] Boyle, A. K. , S. F. Rinaldi , J. E. Norman , and S. J. Stock . 2017. “Preterm Birth: Inflammation, Fetal Injury and Treatment Strategies.” Journal of Reproductive Immunology 119: 62–66. 10.1016/j.jri.2016.11.008. 28122664

[dev70178-bib-0047] Chaalan, F. , F. Minisha , Z. Zaidi , et al. 2024. “Validation of a modified obstetric comorbidity index for prediction of postpartum adverse events including fetal morbidity ‐ a retrospective cohort study from Qatar.” BMC Pregnancy Childbirth 24, no. 1: 415. 38851669 10.1186/s12884-024-06612-x PMC11161943

[dev70178-bib-0006] Chang, Y. , W. Li , Y. Shen , S. Li , and X. Chen . 2023. “Association Between Interleukin‐6 and Preterm Birth: A Meta‐Analysis.” Annals of Medicine 55: 2284384. 10.1080/07853890.2023.2284384. 38010798 PMC10836263

[dev70178-bib-0007] Chavda, V. P. , J. Feehan , and V. Apostolopoulos . 2024. “Inflammation: The Cause of all Diseases.” Cells 13: 1906. 10.3390/cells13221906. 39594654 PMC11592557

[dev70178-bib-0008] Christian, L. M. , R. Glaser , K. Porter , and J. D. Iams . 2013. “Stress‐Induced Inflammatory Responses in Women: Effects of Race and Pregnancy.” Psychosomatic Medicine 75: 658–669. 10.1097/PSY.Ob013e31829bbc89. 23873713 PMC3788648

[dev70178-bib-0009] Côté, F. , E. Prairie , E. M. Sierra , et al. 2025. “A Novel Modulator of IL‐6R Prevents Inflammation‐Induced Preterm Birth and Improves Newborn Outcome.” EMBO Molecular Medicine 17: 1950–1982. 10.1038/s44321-025-00257-9. 40610814 PMC12340070

[dev70178-bib-0010] D'Agata, A. L. , C. Eaton , T. Smith , et al. 2025. “Psychological and Physical Health of a Preterm Birth Cohort at Age 35 Years.” JAMA Network Open 8: e2522599. 10.1001/jamanetworkopen.2025.22599. 40694343 PMC12284743

[dev70178-bib-0048] Easter, S. R. , B. T. Bateman , V. H. Sweeney , et al. 2019. “A comorbidity‐based screening tool to predict severe maternal morbidity at the time of delivery.” American Journal of Obstetrics & Gynecology 221, no. 3: 271.e1–271.e10. 10.1016/j.ajog.2019.06.025 31229427

[dev70178-bib-0011] Engeland, C. G. , J. A. Bosch , and N. Rohleder . 2019. “Salivary Biomarkers in Psychoneuroimmunology.” Current Opinion in Behavioral Sciences 28: 58–65. 10.1016/j.cobeha.2019.01.007. 32215283 PMC7094032

[dev70178-bib-0012] Entringer, S. , K. de Punder , J. Overfeld , et al. 2020. “Immediate and Longitudinal Effects of Maltreatment on Systemic Inflammation in Young Children.” Development and Psychopathology 32: 1725–1731. 10.1017/S094579420001686. 33427162

[dev70178-bib-0013] Giurgescu, C. , C. G. Engeland , T. N. Templin , S. N. Zenk , M. D. Koenig , and L. Garfield . 2016. “Racial Discrimination Predicts Greater Systemic Inflammation in Pregnant African American Women.” Applied Nursing Research 32: 98–103. 10.1016/j.apnr.2016.06.008. 27969060 PMC5159450

[dev70178-bib-0014] Gunnar, M. R. , B. M. Reid , B. Donzella , et al. 2021. “Validation of an Online Version of the Trier Social Stress Test in a Study of Adolescents.” Psychoneuroendocrinology 125: 105111. 10.1016/j.psyneuen.2020.10511. 33341502 PMC7904651

[dev70178-bib-0015] Habelrih, T. , T.‐L. Augustin , F. Mauffette‐Whyte , et al. 2024. “Inflammatory Mechanisms of Preterm Labor and Emerging Anti‐Inflammatory Interventions.” Cytokine & Growth Factor Reviews 78: 50–63. 10.1016/j.cytogfr.2024.07.007. 39048393

[dev70178-bib-0016] Han, V. X. , S. Patel , H. F. Jones , et al. 2021. “Maternal Acute and Chronic Inflammation in Pregnancy Is Associated With Common Neurodevelopmental Disorders: A Systematic Review.” Translational Psychiatry 11: 71. 10.1038/s41398-021-01198-w. 33479207 PMC7820474

[dev70178-bib-0017] Harjunmaa, U. , R. Doyle , J. Järnstedt , et al. 2018. “Periapical Infection May Affect Birth Outcomes via Systemic Inflammation.” Oral Diseases 24: 847–855. 10.1111/odi.12817. 29230915

[dev70178-bib-0018] Harmon, A. C. , D. C. Cornelius , L. M. Amaral , et al. 2016. “The Role of Inflammation in the Pathology of Preeclampsia.” Clinical Science 130: 409–419. 10.1042/CS20150702. 26846579 PMC5484393

[dev70178-bib-0019] Huffhines, L. , Y. Jackson , A. McGuire , and H. M. C. Schreier . 2021. “The Intergenerational Interplay of Adversity on Salivary Inflammation in Young Children and Caregivers.” Psychoneuroendocrinology 128: 105222. 10.1016/j.psyneuen.2021.10522. 33878600 PMC8131264

[dev70178-bib-0020] Jones, S. A. , T. Takeuchi , D. Aletaha , J. Smolen , E. H. Choy , and I. McInnes . 2018. “Interleukin 6: The Biology Behind the Therapy.” Considerations in Medicine 2: 2–6. 10.1136/conmed-2018-000005.

[dev70178-bib-0021] Kirschbaum, C. , K. M. Pirke , and D. H. Hellhammer . 1993. “The ‘Trier Social Stress Test’—A Tool for Investigating Psychobiological Stress Responses in a Laboratory Setting.” Neuropsychobiology 28: 76–81. 10.1159/000119004. 8255414

[dev70178-bib-0022] Laurent, H. K. , S. H. Goodman , D. A. Granger , and P. M. Backer . 2025. “Coordination of Inflammatory and Adrenocortical Stress Response During Pregnancy and Variation by Mental Health.” Brain, Behavior, & Immunity ‐ Health 50: 101145. 10.1016/j.bbih.2025.101145. PMC12686803 41378190

[dev70178-bib-0023] Lengacher, C. A. , R. R. Reich , C. L. Paterson , et al. 2018. “A Large Randomized Trial: Effects of Mindfulness‐Based Stress Reduction (MBSR) for Breast Cancer (BC) Survivors on Salivary Cortisol and IL‐6.” Biological Research for Nursing 21: 39–49. 10.1177/1099800418789777. 30079756 PMC6700883

[dev70178-bib-0024] Martinez, A. D. , L. Ruelas , and D. A. Granger . 2018. “Household Fear of Deportation in Relation to Chronic Stress and Salivary Proinflammatory Cytokines in Mexican‐Origin Families Post‐SB‐1070.” Social Science Medicine Population Health 5: 188–200. 10.1016/j.ssmph.2018.06.003. PMC6068082 30073186

[dev70178-bib-0025] Méndez Leal, A. S. , J. A. Silvers , J. E. Carroll , et al. 2023. “Maternal Early Life Stress Is Associated With Pro‐Inflammatory Processes During Pregnancy.” Brain, Behavior, and Immunity 109: 285–291. 10.1016/j.bbi.2022.10.012. 36280180 PMC10035632

[dev70178-bib-0026] Mitchell, A. M. , K. Porter , and L. M. Christian . 2018. “Examination of the Role of Obesity in the Association Between Childhood Trauma and Inflammation During Pregnancy.” Health Psychology 37: 114–124. 10.1037/hea0000559. 28967771 PMC5794605

[dev70178-bib-0027] Moog, N. K. , S. Nolvi , T. S. Kleih , et al. 2021. “Prospective Association of Maternal Psychosocial Stress in Pregnancy With Newborn Hippocampal Volume and Implications for Infant Social‐Emotional Development.” Neurobiology of Stress 15: 100368. 10.1016/j.ynstr.2021.100368. 34355050 PMC8319845

[dev70178-bib-0028] Mor, G. , P. Aldo , and A. B. Alvero . 2017. “The Unique Immunological and Microbial Aspects of Pregnancy.” Nature Reviews Immunology 17: 469–482. 10.1038/nri.2017.64. 28627518

[dev70178-bib-0029] Moreno, J. J. 2024. “Modulation of Inflammatory Response and Pain by Mind‐Body Therapies as Meditation.” Brain Behavior and Immunity Integrative 5: 100036. 10.1016/j.bbii.2023.1000036.

[dev70178-bib-0030] Riis, J. L. , H. Ahmadi , K. R. Hamilton , T. Hand , and D. A. Granger . 2021. “Best Practice Recommendations for the Measurement and Interpretation of Salivary Proinflammatory Cytokines in Biobehavioral Research.” Brain, Behavior, and Immunity 91: 105–116. 10.1016/j.bbi.2020.09.009. 32931871 PMC8164445

[dev70178-bib-0031] Robinson, D. P. , and S. L. Klein . 2012. “Pregnancy and Pregnancy‐Associated Hormones Alter Immune Responses and Disease Pathogenesis.” Hormones and Behavior 62: 263–271. 10.1016/j.yhbeh.2012.02.023. 22406114 PMC3376705

[dev70178-bib-0032] Rudolph, M. D. , A. M. Graham , E. Feczko , et al. 2018. “Maternal IL‐6 During Pregnancy Can Be Estimated From Newborn Brain Connectivity and Predicts Future Working Memory in Offspring.” Nature Neuroscience 21: 765–772. 10.1038/s41593-018-0128-y. 29632361 PMC5920734

[dev70178-bib-0033] Sanada, K. , J. Montero‐Marin , A. Barceló‐Soler , et al. 2020. “Effects of Mindfulness‐Based Interventions on Biomarkers and Low‐Grade Inflammation in Patients With Psychiatric Disorders: A Meta‐Analytic Review.” International Journal of Molecular Sciences 21: 2484. 10.3390/ijms21072484. 32260096 PMC7177919

[dev70178-bib-0034] Scholaske, L. , A. Brose , J. Spallek , and S. Entringer . 2019. “Perceived Discrimination and Risk of Preterm Birth Among Turkish Immigrant Women in Germany.” Social Science & Medicine 236: 112427. 10.1016/j.socscimed.2019.112427. 31352314 PMC7327293

[dev70178-bib-0035] Schreier, H. M. C. , Y. I. Kuras , C. M. McInnis , et al. 2020. “Childhood Physical Neglect Is Associated With Exaggerated Systemic and Intracellular Inflammatory Responses to Repeated Psychosocial Stress in Adulthood.” Frontiers in Psychiatry 11: 504. 10.3389/fpsyt.2020.00504. 32581878 PMC7290130

[dev70178-bib-0036] Segerstrom, S. C. , and G. E. Miller . 2004. “Psychological Stress and the Human Immune System: A Meta‐Analytic Study of 30 Years of Inquiry.” Psychological Bulletin 130: 601–630. 10.1037/0033-2909.130.4.601. 15250815 PMC1361287

[dev70178-bib-0037] Shonkoff, J. P. , A. S. Garner , Committee on Psychosocial Aspects of Child and Family Health, et al. 2012. “The Lifelong Effects of Early Childhood Adversity and Toxic Stress.” Pediatrics 129: e232–e246. 10.1542/peds.2011-2663. 22201156

[dev70178-bib-0038] Slavish, D. C. , J. E. Graham‐Engeland , J. M. Smyth , and C. G. Engeland . 2015. “Salivary Markers of Inflammation in Response to Acute Stress.” Brain, Behavior, and Immunity 44: 253–269. 10.1016/j.bbi.2014.08.008. 25205395 PMC4275319

[dev70178-bib-0039] Tollenaar, M. S. M. , K. K. Pittner , R. S. M. R. Buisman , et al. 2022. “Salivary Immune Markers Are Not Associated With Self‐Reported Childhood Maltreatment or Psychopathology in Adults.” Psychoneuroendocrinology 144: 105867. 10.1016/j.psyneuen.2022.105867. 35863154

[dev70178-bib-0040] Tsikouras, P. , E. Oikonomou , K. Nikolettos , et al. 2024. “The Impact of Periodontal Disease on Preterm Birth and Preeclampsia.” Journal of Personalized Medicine 14: 345. 10.3390/jpm14040345. 38672972 PMC11051368

[dev70178-bib-0041] Tsuyuki, K. , A. N. Cimino , C. N. Holliday , J. C. Campbell , N. A. Al‐Alusi , and J. K. Stockman . 2019. “Physiological Changes From Violence‐Induced Stress and Trauma Enhance HIV Susceptibility Among Women.” Current HIV/AIDS Reports 16: 57–65. 10.1007/s11904-019-00435-8. 30762216 PMC6420839

[dev70178-bib-0042] Tyrka, A. R. , S. H. Parade , T. R. Valentine , N. M. Eslinger , and R. Seifer . 2015. “Adversity in Preschool‐Aged Children: Effects on Salivary Interleukin‐1β.” Development and Psychopathology 27: 567–576. 10.1017/S0954579415000164. 25997772 PMC4877166

[dev70178-bib-0043] Wadhwa, P. D. , S. Entringer , C. Buss , and M. C. Lu . 2012. “The Contribution of Maternal Stress to Preterm Birth: Issues and Considerations.” Clinics in Perinatology 38: 351–384. 10.1016/j.clp.2011.06.007. PMC3179976 21890014

[dev70178-bib-0044] Walsh, K. , A. Basu , E. Werner , et al. 2016. “Associations Among Child Abuse, Depression, and Interleukin‐6 in Pregnant Adolescents: Paradoxical Findings.” Psychosomatic Medicine 78: 920–930. 10.1097/PSY.0000000000000344. 27187846 PMC5067964

[dev70178-bib-0045] Wong, C. , S. Patel , A. LaPorta , et al. 2023. “Correlation Analysis of Salivary Cytokines and Hormones With Resiliency.” Journal of Trauma and Acute Care Surgery 95: 664–671. 10.1097/TA.0000000000004026. 37332103 PMC10637304

[dev70178-bib-0046] Yui, S. , D. Sasayama , M. Yamaguchi , and S. Washizuka . 2022. “Altered Levels of Salivary Cytokines in Patients With Major Depressive Disorder.” Clinical Neurology and Neurosurgery 221: 107390. 10.1016/j.clineuro.2022.107390. 35917728

